# Using Metabolomics to Identify Cell Line-Independent Indicators of Growth Inhibition for Chinese Hamster Ovary Cell-Based Bioprocesses

**DOI:** 10.3390/metabo10050199

**Published:** 2020-05-15

**Authors:** Nicholas Alden, Ravali Raju, Kyle McElearney, James Lambropoulos, Rashmi Kshirsagar, Alan Gilbert, Kyongbum Lee

**Affiliations:** 1 Department of Chemical and Biological Engineering, Tufts University, 4 Colby Street, Medford, MA 02155, USA; nalden246@gmail.com; 2 Biogen, 225 Binney St, Cambridge, MA 02142, USA; ravali.raju@gmail.com (R.R.); kyle.mcelearney@gmail.com (K.M.); james.lambropoulos@biogen.com (J.L.); Rashmi.kshirsagar@rubiustx.com (R.K.); alanbgilbert@gmail.com (A.G.)

**Keywords:** Chinese hamster ovary cell, untargeted metabolomics, tryptophan, 5-hydroxyindoleacetaldehyde, growth inhibitor

## Abstract

Chinese hamster ovary (CHO) cells are widely used for the production of biopharmaceuticals. Efforts to improve productivity through medium design and feeding strategy optimization have focused on preventing the depletion of essential nutrients and managing the accumulation of lactate and ammonia. In addition to ammonia and lactate, many other metabolites accumulate in CHO cell cultures, although their effects remain largely unknown. Elucidating these effects has the potential to further improve the productivity of CHO cell-based bioprocesses. This study used untargeted metabolomics to identify metabolites that accumulate in fed-batch cultures of monoclonal antibody (mAb) producing CHO cells. The metabolomics experiments profiled six cell lines that are derived from two different hosts, produce different mAbs, and exhibit different growth profiles. Comparing the cell lines’ metabolite profiles at different growth stages, we found a strong negative correlation between peak viable cell density (VCD) and a tryptophan metabolite, putatively identified as 5-hydroxyindoleacetaldehyde (5-HIAAld). Amino acid supplementation experiments showed strong growth inhibition of all cell lines by excess tryptophan, which correlated with the accumulation of 5-HIAAld in the culture medium. Prospectively, the approach presented in this study could be used to identify cell line- and host-independent metabolite markers for clone selection and bioprocess development.

## 1. Introduction

Chinese hamster ovary (CHO) cells are among the most widely used production hosts for biopharmaceuticals, especially monoclonal antibodies (mAbs), due to their capacity to support proper protein folding and post-translational modifications that are critical for therapeutic efficacy [1]. Advances in process control, medium formulation, and host cell engineering have dramatically increased the volumetric productivity of CHO cell lines [2], with titers for some products reaching 10 grams per liter or higher [3].

Despite progress, limitations remain with respect to productivity. One major source of productivity limitation in fed-batch bioreactors is the accumulation of metabolic byproducts that reduce cell growth, viability and/or protein production [4]. Controlling these byproducts, notably lactate and ammonia, has in some cases substantially improved the performance of CHO cell-based bioprocesses [5]. In recent years, other metabolites have been found to accumulate in CHO cell cultures that negatively correlate with growth and/or productivity [6]. These accumulating metabolites include intermediates or byproducts of not only glycolysis and the TCA cycle, but also other pathways, including amino acid, nucleotide, lipid, and redox metabolism [7,8,9]. These findings show that inefficiencies can occur in many different parts of metabolism, and suggest that an omics approach is warranted to more broadly and systematically investigate the metabolic byproducts of industrially relevant CHO cell cultures [6,10].

Metabolomics experiments generally fall into two categories—targeted or untargeted. Targeted experiments focus on a selected subset of the metabolome to obtain quantitative measurements on these metabolites. Untargeted experiments provide a semi-quantitative, but more comprehensive profile of metabolites in a biological system. These experiments are performed without a priori selection of target analytes, and thus provide a useful means of reducing bias for discovery oriented studies [11].

In the context of CHO cell-based bioprocesses, untargeted metabolomics has been used to examine the effects of growth medium composition [12,13] and inform rational medium design [14,15]. A handful of studies have used metabolomics experiments to compare the metabolic characteristics of high-growth or high-productivity cell lines, with the aim of identifying metabolites that accumulate in the culture and inhibit growth and/or productivity [8,16]. These previous studies have typically focused on a single parental strain, or “host” (e.g., CHO-K1 or GS-CHO). As a result, it is unclear whether the metabolites identified in these studies are only relevant to a particular cell line from a specific host. Identifying more general indicators of growth and/or productivity would be valuable for developing platform processes compatible with multiple hosts, e.g., during clone selection, as this would help relieve the burden of separately designing a specialized culture medium and feeding strategy for each new product molecule.

In this paper, we describe an untargeted analysis of six CHO cell lines derived from two hosts, with each cell line producing a different mAb. Comparisons of accumulating metabolites across cell lines as well as between different growth phases (the exponential growth phase vs. the stationary phase) point to a tryptophan-derived metabolite as an indicator of significant growth inhibition. Supplementation experiments confirmed that excess tryptophan impairs the growth of multiple CHO cell lines from different hosts.

## 2. Results

### 2.1. Metabolic Profiles of Cell Lines Depend on Lineage and Growth Characteristics

**Figure 1:**
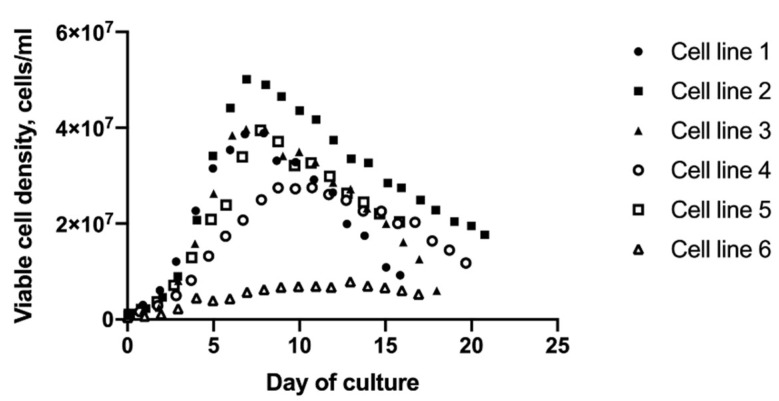
Daily measurements of viable cell density. Cell lines from host 1 (CHO K1 GS knockout) and host 2 (DG44) are indicated by closed and open symbols, respectively. Each cell line produces a different mAb. All six cell lines were grown using the same basal and feed media under identical reactor operating conditions.

Six CHO cell lines producing different mAbs were selected to investigate the relationship between metabolite profile and cell growth. The six cell lines were derived from two different parental cell lines, or hosts, and selected based on their growth characteristics. The cell lines were cultured under identical bioreactor conditions but grew at different rates. The peak VCDs ranged from 7.8 to 50.1 × 10^6^ cells/mL (Figure 1). Cell line 2 from host 1 and cell line 6 from host 2 exhibited the highest and lowest growth rates, respectively. The growth rates were not strictly host dependent, as the other four cell lines reached similar peak VCDs between days 7 and 9. To obtain a global profile of metabolites for each cell line at different growth stages, culture medium samples were collected while the cultures were in exponential growth (the exponential growth phase) and when net growth had peaked (the stationary phase) prior to the cultures entering a period of decline in VCD. The samples were clarified by centrifugation to remove cells and analyzed using untargeted LC–MS experiments.

**Figure 2:**
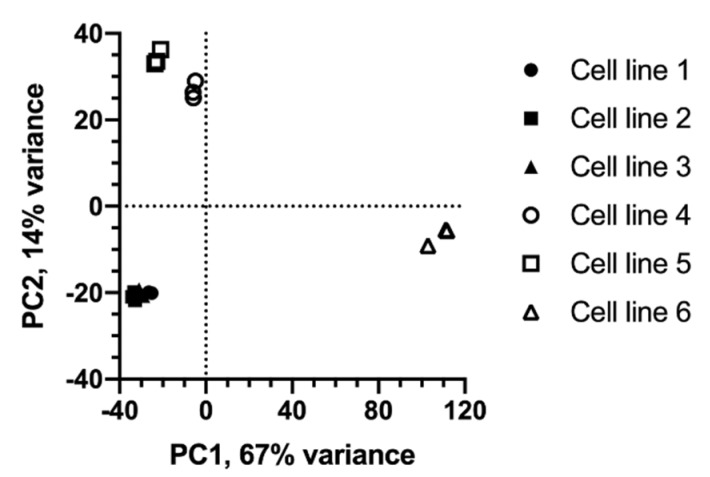
Scatter plot of principal component (PC) scores from global metabolite profiles of culture supernatant samples collected at peak viable cell density. Cell lines from CHO K1 GS knockout- and DG44-derived hosts are indicated by closed and open symbols, respectively. Each plot marker represents one of three parallel extractions for a sample. Principal component analysis was performed on autoscaled LC–MS data, i.e., responses associated with the feature.

Principal component analysis (PCA) of autoscaled LC–MS data from samples collected during the stationary phase shows distinct groupings of cell lines (Figure 2). A scatter plot of the first two principal component (PC1 and PC2) scores shows that the cell lines group based on both growth characteristics and host cell lineage. All three cell lines from host 1 group closely together in the lower left quadrant (negative PC1 and PC2 scores). In comparison, the cell lines from host 2 project further apart. The projection of a cell line along PC1 reflects its peak VCD. Cell line 2 reached the highest peak VCD, and projects furthest to the left (PC1 score = −33). Cell lines 1, 3 and 5 reached the next highest peak VCDs, and have PC1 scores between −29 and −23. Cell line 4 reached an intermediate peak VCD, and has a PC1 score of −6. Cell line 6 grew more slowly to a substantially lower peak VCD (~16% of cell line 2) than the other five cell lines, and has a PC1 score of 109. It is worth noting that cell lines 1 and 3 (host 1) and cell line 5 (host 2) grew to a similar peak VCD and have a similar PC1 score even though they are derived from two different hosts. Along PC2, cell lines from host 2 have higher scores than host 1 irrespective of peak VCDs. A similar separation of cell lines based on host lineage and growth was observed when the data were log-transformed (not shown). These observations suggested that the differences in metabolite profiles between cell lines depend on the cell lines’ host lineage as well as growth characteristics.

### 2.2. Tryptophan Metabolism Negatively Correlates with Growth

To determine whether the observed growth differences are significantly associated with specific metabolic activities, pathway enrichment analysis was performed on metabolite profiles of stationary phase samples using KEGG pathway maps for the Chinese hamster as reference. Pathways represented by a higher than expected number of metabolites, as determined by a modified Fisher’s exact test, were considered “enriched” in the low-growth cell line 6. The results of this analysis are shown in Table 1. Aminoacyl-tRNA biosynthesis has the lowest FDR-adjusted *p*-value. However, the significance of this pathway reflects its membership of all 20 naturally occurring amino acids and their cognate tRNAs. Therefore, we focused our subsequent analysis on the other two significant pathways—histidine and tryptophan metabolism.

**Figure 3:**
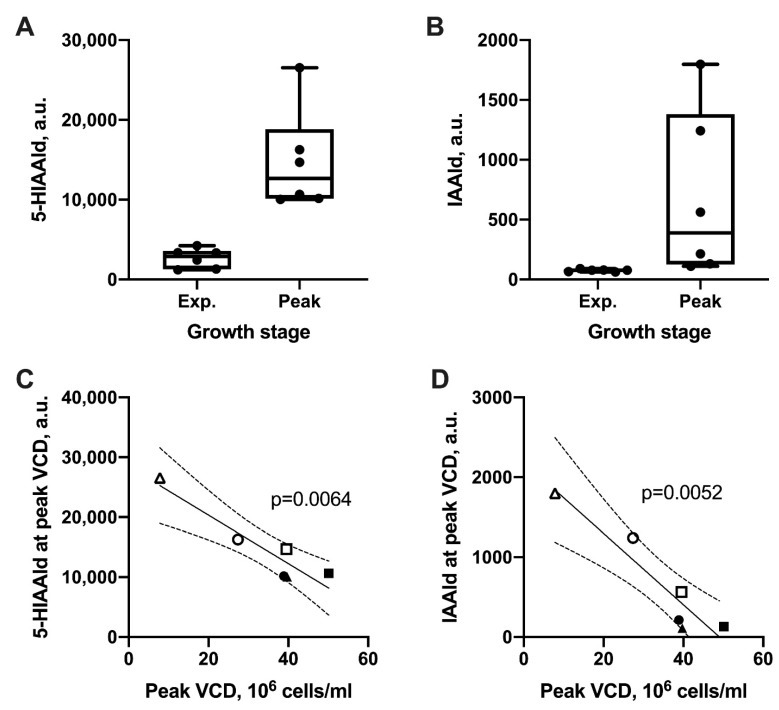
Tryptophan metabolites negatively correlating with cell growth. Medium concentrations of LC–MS features putatively identified as (**A**) 5-hydroxyindoleacetaldehyde (5-HIAAld) and (**B**) indole 3-acetaldehyde (IAAld) during the exponential growth phase and the stationary phase in the bioreactor cultures. Box plots show the maximum, minimum, and median responses of the LC–MS features (areas-under-the-curve in extracted ion chromatograms) across the six cell lines. (**C**,**D**) Scatter plots showing regression lines and 95% confidence bands for (**C**) 5-HIAAld and (**D**) IAAld levels in the culture medium at peak VCDs of the six cell lines. Cell lines from host 1 (CHO K1 GS knockout) and host 2 (DG44) are indicated by closed and open symbols, respectively. A *p*-value < 0.05 indicates that the regression line slope is significantly different than zero by F-test.

We next sought to identify metabolites that accumulated in all six cell lines as the cultures transitioned from the exponential growth phase to the stationary phase, while also negatively correlating with peak VCD. We performed a two-way ANCOVA with growth stage and peak VCD as the two factors. This analysis identified 367 significant features (FDR-adjusted *p*-value < 0.05) that meet these criteria (Figure S1). Of these, 179 were significantly elevated during the stationary phase and were inversely correlated with peak VCD. Using BioCAn, putative metabolite identities were assigned to 11 of these features (Table S1). Two of these 11 metabolites belong to tryptophan metabolism: 5-hydroxyindolacetaldehyde (5-HIAAld, an oxidation product of serotonin, Figure 3A) and indole-3-acetaldehyde (IAAld, an oxidation product of tryptamine, Figure 3B). Another significantly accumulating metabolite was putatively annotated as N-formimino-L-glutamate, which can be formed from histidine or glutamate (Figure S2). Interestingly, the bioreactor cultures did not show a significant correlation between tryptophan itself and peak VCD for the six cell lines (Figure S3). Further, we did not detect any significant differences in tryptophan concentrations between the exponential growth phase and the stationary phase of the six cell lines. Taken together, these results suggested that one or more products of tryptophan metabolism could play a role in the inhibition of cell growth.

### 2.3. Excess Tryptophan Inhibits the Growth of Multiple Cell Lines From Different Hosts

**Figure 4:**
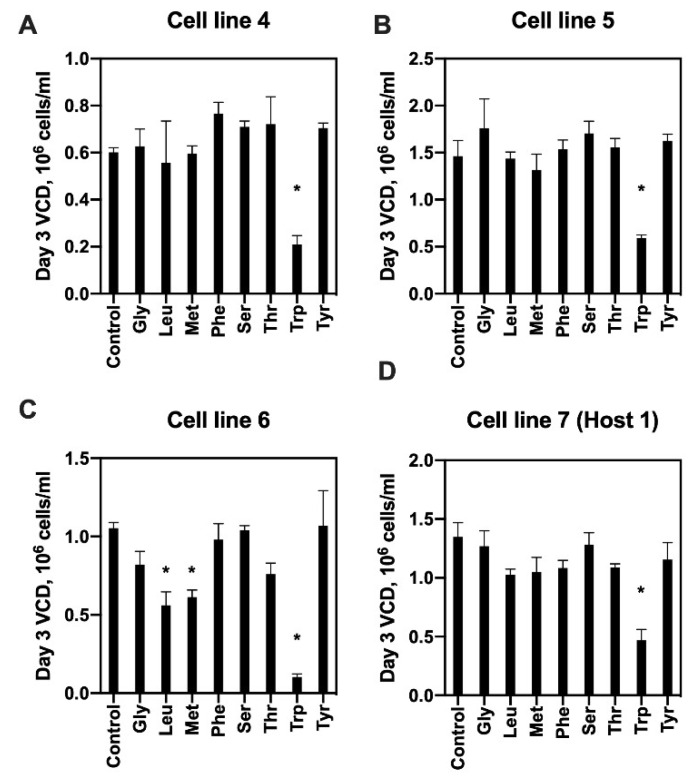
Effects of amino acid supplementation on cell growth. Host 2-derived cell lines 4–6 (**A**–**C**) and host 1-derived cell line 7 (**D**) were cultured in basal medium (control) or basal medium supplemented with one of eight amino acids at 10× their basal medium concentration. Each culture was carried out in duplicate. Bar graph values show viable cell density (VCD) measured on day 3. *: Benjamini–Hochberg procedure-adjusted ANOVA *p*-value < 0.1.

To determine whether tryptophan could directly inhibit the growth of any of the six cell lines from the bioreactor experiments, a cell line (cell line 4 from host 2) that grew to an intermediate peak VCD (halfway between cell lines 2 and 6) was cultured in shake flasks with varying levels of tryptophan (1×, 5× and 10× of basal medium concentration). We observed a significant negative correlation between the specific growth rate on day 3 and level of tryptophan supplementation (ANOVA *p*-value = 0.012, Figure S4). Culturing the cells at 10× tryptophan concentration reduced the VCD by nearly 60% on day 3. Based on this result, we next investigated whether excess tryptophan similarly inhibits the growth of other cell lines and whether other amino acids also have this effect. To this end, a similar supplementation experiment was performed on cell lines 4–6 from host 2 and a new cell line from host 1, designated as cell line 7 (Figure 4). Cell line 4 was included in these experiments to verify that growth inhibition by excess tryptophan also occurs in the deep-well culture format. Cell lines 5 and 6 were included to test the other two cell lines from the same host that showed, respectively, higher and lower peak VCD. Cell line 7 was included to test whether the inhibition by excess tryptophan also occurs in a “new” cell line that had not been used to identify the phenomenon. A total of eight additional amino acids were tested in 24 deep-well plate cultures. These amino acids were selected based on a recent study [8] that reported growth inhibitory effects of their metabolic byproducts. All four cell lines showed significant growth inhibition at 10× tryptophan concentration above the basal level, with the fold reduction in day 3 VCD ranging from 10.3 (cell line 6) to 2.5 (cell line 5). None of the other amino acids showed a similar degree of growth inhibition for any of the cell lines. Increasing methionine or leucine to 10× above the basal level led to a significant, but less severe growth inhibition (a 1.7- to 1.9-fold reduction in day 3 VCD) for cell line 6. These results suggested that tryptophan or another metabolite that accumulated in the culture due to excess tryptophan broadly inhibits the growth of mAb-producing CHO cells.

### 2.4. Tryptophan-Derived Metabolite Is a Potential Indicator of Growth Inhibition

**Figure 5:**
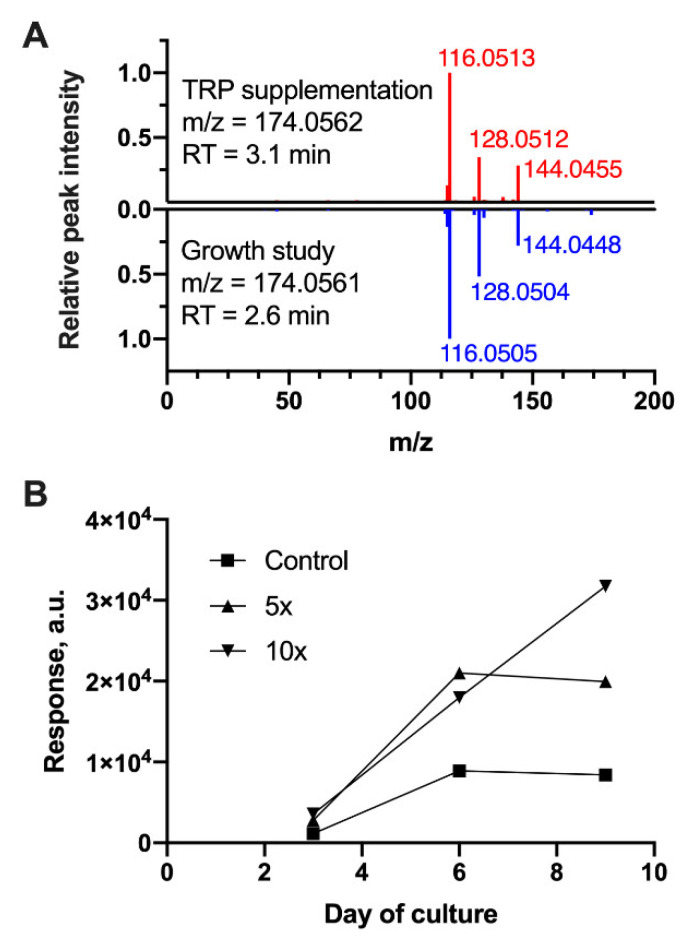
Dose-dependent accumulation of an LC–MS feature in tryptophan (TRP)-supplemented shake flask culture. (**A**) Mirror plot of MS/MS spectrum for the feature detected in TRP-supplemented shake-flask cultures (top spectrum, red peaks) and the feature annotated as 5-hydroxyindoleacetaldehyde (5-HIAAld) in bioreactor cultures (bottom spectrum, blue peaks). *m/z*: mass-to-charge ratio of precursor ion recorded in the negative ionization mode. RT: chromatographic retention time. (**B**) Response profile of the LC–MS feature shown in the top spectrum of panel (**A**). Response refers to the feature’s area under the curve in extracted ion chromatogram and represents the corresponding metabolite’s concentration in the culture medium. The level of TRP supplementation above the basal medium concentration (control) is indicated in the figure legend. Data shown are the mean ± SD of duplicate cultures. Error bars are smaller than plot symbols.

We next profiled the metabolites in culture medium samples from the shake flask supplementation experiments to determine whether either of the two putatively identified tryptophan metabolites (5-HIAAld and IAAld) accumulated in the shake flask cultures with increasing tryptophan concentration. The samples from these cultures were analyzed using the same LC–MS experiments as the bioreactor study. Analysis of variance identified 112 LC–MS features with responses that significantly associate with tryptophan concentration (FDR-adjusted *p*-value < 0.05). Of these, eight features were annotated by BioCAn as tryptophan metabolites per KEGG’s pathway definition. These features included 5-HIAAld, but not IAAld (Table S4). Further, the RT and MS/MS spectrum of the feature annotated as 5-HIAAld exactly matched the corresponding feature from the original growth study (Figure 5A). Figure 5B shows the accumulation of this feature in the culture medium. In the 10× conditions, the feature continued to accumulate through day 9, whereas it reached a plateau in the basal and 5× conditions, suggesting that accumulation is driven by tryptophan availability.

**Figure 6:**
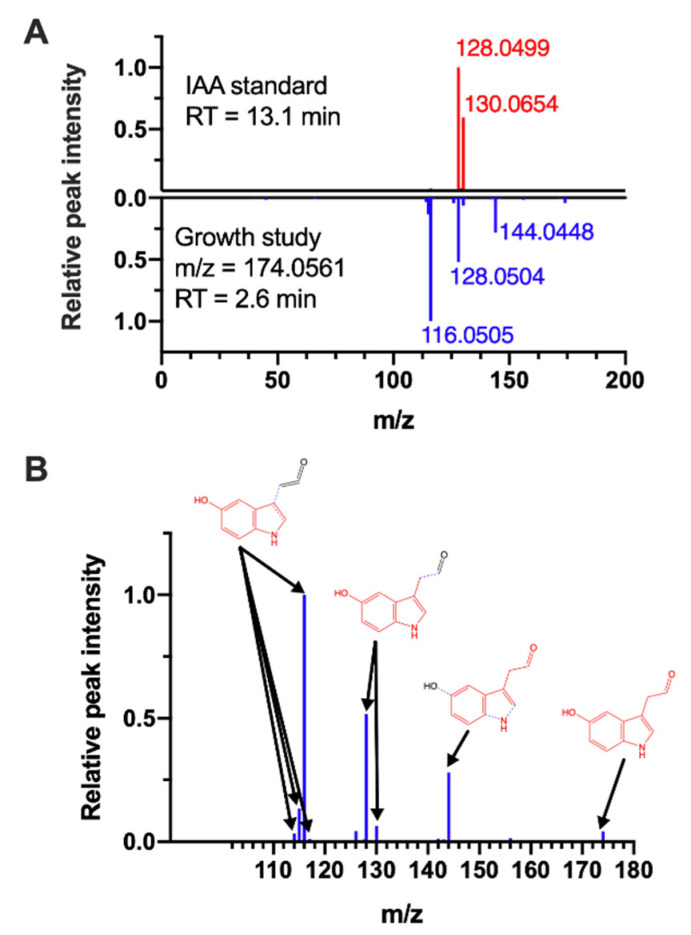
Annotation of 5-hydroxyindoleacetaldehyde (5-HIAAld). (**A**) Mirror plot of MS/MS spectrum for indole 3-acetate standard (top spectrum, red peaks) and the feature putatively identified as 5-HIAAld (bottom spectrum, blue peaks). The standard was run on the same instrument using the same LC–MS method as the sample. (**B**) Fragment structures of 5-HIAAld assigned by NIST MS Interpreter to the MS/MS spectrum shown in panel (**A**). Arrows point from fragments and ionization-related modifications (protonation, deprotonation, hydrogen transfer, and/or loss of water) to corresponding ion peaks. The fragments are highlighted in red. Dotted blue lines indicate covalent bonds that are broken to generate the fragment.

The annotation tool used in this study, BioCAn, assigns putative identities to the detected features. Confirming an annotation requires an authentic chemical standard. Unfortunately, 5-HIAAld is unavailable for purchase from a commercial supplier. We thus tested whether the feature of interest could match another mammalian metabolite with the same exact mass as 5-HIAAld. To account for the possibility that such a metabolite is missing in our CHO cell model due to incomplete annotation of the Chinese hamster genome, we included models of mouse, rat, human, and rhesus macaque in this analysis. Across all five models, the only other metabolite with a matching exact mass is indole-3-acetic acid (IAA). For IAA, a high-purity chemical standard is readily available. Figure 6A shows a mirror plot comparing the MS/MS spectrum of the feature annotated as 5-HIAAld against the MS/MS spectrum for the IAA standard analyzed on the same instrument under identical conditions. This analysis clearly eliminates IAA as a possible identity for the feature of interest. We next sought to determine whether the MS/MS spectrum masses observed for the feature of interest could be explained by collision-induced fragmentation of 5-HIAAld. Using MS Interpreter (NIST version BETA 3.1a), we investigated possible structural origins of the MS/MS peaks for the feature of interest, and found that ion fragments and neutral ion losses of 5-HIAAld explain 9 out of the 11 peaks (Figure 6B).

## 3. Discussion

Using untargeted LC–MS experiments, we correlated the growth of mAb-producing CHO cells cultured in fed-batch reactors with metabolites that accumulated in the culture medium. A novel aspect of the study is that the correlation was identified across multiple cell lines derived from two different hosts, with each cell line producing a different mAb. We found a significant negative correlation between peak VCD and a metabolite annotated as an intermediate in tryptophan metabolism. This finding is supported by results from medium supplementation experiments, which confirmed a growth inhibitory effect of excess tryptophan. Importantly, this effect was observed for multiple cell lines, including a cell line that was not included in the bioreactor study that identified the negative correlation between peak VCD and tryptophan metabolism. We annotated the tryptophan-derived metabolite as 5-HIAAld based on all available evidence. As 5-HIAAld is unavailable for purchase, we were unable to directly determine its effect on cell growth by adding it to the culture medium. However, we show that a metabolite with a spectral signature matching 5-HIAAld accumulates with increasing tryptophan concentration in the culture medium. We also show that this metabolite cannot be IAA, the only other CHO cell metabolite with nearly the same mass (within 0.003 Da of 5-HIAAld), as the RT and MS/MS spectrum clearly do not match (Figure 6A). The MS/MS fragmentation analysis using MS Interpreter further supports that 5-HIAAld is likely the correct identity for the metabolite (Figure 6B). These results provide the first direct evidence of growth inhibition by excess tryptophan, and suggest that 5-HIAAld (or another unknown metabolite matching the mass, RT and predicted fragmentation spectrum of 5-HIAAld) is a cell line- and host-independent indicator of growth inhibition for mAB-producing CHO cells.

Several recent studies have used metabolomics experiments to investigate growth limitations in CHO cell-based bioprocesses. Mulukutla et al. reported that several amino acids and related metabolites, including tryptophan derivatives indole 3-carboxylate and indole 3-lactate, accumulated in fed-batch cultures of a glutamine synthetase (GS) knockout CHO cell line [8]. Even though these cultures controlled the accumulation of lactate using the HiPDOG feeding strategy [5], growth eventually slowed, which the study attributed to the accumulation of metabolic byproducts. The study also found that maintaining the amino acids in the culture medium at a low level resulted in an increase in VCD and protein titer.

In addition to discovering growth inhibitory byproducts, untargeted metabolomics has also been used to improve medium supplementation strategies. For example, Sellick et al. used GC–MS-based metabolite profiling experiments to find that glucose and several amino acids become depleted at different time points in culture, and that supplementing the feed medium with these nutrients increased peak VCD and maximal titer [15]. More recently, Chong et al. compared the metabolite profiles of several clones that produced the same mAb and were derived from the same host lineage (CHO DG44), but exhibited different specific productivities [16]. The study reported positive associations between specific productivity, redox metabolites and activated sugars; however, the study did not include an add-back experiment to confirm whether these metabolites directly impact productivity. Using untargeted GC–MS experiments and HPLC assays, Dietmair et al. evaluated the impact of commercial culture media formulations on the growth characteristics of a human growth hormone producing CHO cell line [12]. While the number of metabolites profiled was relatively small, the study identified several nucleotides and amino acids positively associated with high growth rates.

A common denominator of the above studies is that growth and productivity correlate with intermediates of not only glucose catabolism, but also amino acid metabolism. However, because each study examined a different cell line under varying culture conditions, it is unclear whether any general conclusions can be drawn that are applicable to other hosts and cell lines. The present study takes a step towards addressing this issue by comparing multiple cell lines derived from different hosts that were grown under identically controlled bioreactor conditions. Our supplementation experiments demonstrate that excess tryptophan, but not other amino acids, impairs the growth of cell lines derived from hosts of two different strains (K1 and DG44). This result points to the possibility that cell lines with different genetic backgrounds could share a common mechanism of growth inhibition.

A major challenge in metabolomics is metabolite identification, where problems persist regarding low annotation rates, ambiguity, and inconsistency of annotation across studies. There are several factors that contribute to these problems. For a given metabolite, even the largest spectral libraries catalog a handful of experimentally obtained mass features, where the experimental conditions for the library data may or may not match the conditions for the study of interest. Ideally, metabolite annotations are confirmed using high-purity chemical standards run on the same instrument under identical experimental conditions, but these standards are unavailable and too costly to synthesize for many metabolites. Another factor is data preprocessing, which impacts both the quantity of unique mass features that can be extracted from an untargeted experiment as well as the information content of these features. In a recent study, Yeo et al. showed that certain ion products (sometimes termed adducts) of soft (ESI) ionization are highly specific for some subclasses of lipids, and that associating an ion product with the most likely source lipid molecule based on a dominant, preferred ion product of the lipid during preprocessing could improve both the coverage and accuracy of subsequent metabolite annotation [17].

In the present study, we analyzed the annotations for a detected compound suggested by different tools in the context of known enzymatic reactions for the CHO cell (Supplementary Materials). This approach ensured that an LC–MS feature is only annotated if it is a reactant or product of a CHO cell enzyme. If the mass of a detected compound matches more than one metabolite, then our annotation method resolves the ambiguity by determining which metabolite is more likely to be present in the sample based on the number of other biochemically connected metabolites that are also detected. Using this method, we determined that the tryptophan metabolite indicative of growth inhibition is more likely to be 5-HIAAld than IAA (BioCAn annotation scores of 1.93 and 0.52 respectively). This is further supported by the finding that the RT and MS/MS spectrum of IAA does not match the metabolite of interest (Figure 6A). Clearly, our annotation results depend on the accuracy and completeness of the underlying metabolic model. The present study used a model of CHO cell metabolism assembled from Chinese hamster reactions cataloged in KEGG [18], which we found to provide a very similar level of accuracy to the *i*CHO model [19] downloaded from the BiGG database [20]. We also compared the KEGG-based model against an up-to-date, curated model by Calemels et al. [21] to ensure that we did not omit CHO cell-associated tryptophan metabolites that could better explain the feature annotated as 5-HIAAld.

Whether this tryptophan metabolite directly inhibits CHO cell growth remains to be elucidated. One interpretation of the supplementation experiments is that tryptophan itself is a growth inhibitory metabolite. On the other hand, we did not find a significant negative correlation between tryptophan and peak VCD in the untargeted metabolomics data from the bioreactor experiments (Figure S3). Rather, negative correlations were found with LC–MS data features that were annotated as products of tryptophan metabolism. We hypothesized that these products would accumulate in the culture medium if the cells were fed excess tryptophan. Consistent with this hypothesis, we observed a dose-dependent increase in LC–MS features annotated as indole-containing metabolites upon tryptophan supplementation (Table S3). Only one of these features, annotated as 5-HIAAld, also correlated negatively with growth in the bioreactor experiments. It is possible that this feature does not represent a growth inhibitory metabolite and merely indicates the accumulation of another metabolite that negative impacts cell growth. A previous study [8] suggested that indole-3-lactate, which can be derived from tryptophan, is a CHO cell growth inhibitor. We could not detect this metabolite in either bioreactor or shake flaks cultures using our LC–MS assay, which has a detection limit (1.5 μM) well below the inhibitory concentration (3 mM) reported in the referenced study.

The accumulation of 5-HIAAld could reflect the depletion of an upstream metabolite that promotes cell growth (Figure S5). In murine and human cells, 5-HIAAld is formed from serotonin by monoamine oxidase A (MAO-A), a catecholamine-metabolizing enzyme that is downregulated in human and animal cancer tissues [22]. It has been shown that serotonin suppresses apoptosis in transformed human hepatocytes [23], and that 5-HIAAld is depleted in the plasma of patients with ovarian cancer [24]. To investigate whether depletion of serotonin could explain the negative association between 5-HIAAld and growth, we assessed the impact of serotonin supplementation. This increased the VCD of cell line 7 but had no significant impact on the other three cell lines we tested (Figure S6), suggesting that serotonin depletion is not a general mechanism for CHO cell growth inhibition. An alternative explanation is that tryptophan catabolism produces indole metabolites, which have been reported to reduce cell viability [8]. We found that addition of 50 μM IAA reduced the growth rate of CHO cells by 85% (Figure S7). Like 5-HIAAld, IAA derives from tryptophan and contains an indole moiety. It is possible that the accumulation of indole containing tryptophan catabolites could represent a currently unknown general toxicity mechanism in CHO cells.

Obtaining further insights into whether 5-HIAAld modulates CHO cell growth or merely indicates growth inhibition would benefit from molecular approaches that disrupt specific steps in tryptophan metabolism, for example, using CRISPR/Cas9 mediated gene knockout. This strategy has been successfully demonstrated for lipoprotein lipase, achieving near complete knockout of this enzyme in CHO cells [25]. Prospectively, gene knockout strategies could be used to prevent the formation of harmful byproducts. However, the targets would have to be carefully considered to control for unintended effects; for example, MAO-A can act on a number of other substrates in addition to serotonin [26]. Stable isotopic tracer experiments [27], e.g., using ^13^C- and ^15^N-labeled tryptophan, could be used in conjunction with untargeted metabolomics to characterize the impact of enzyme knockouts (or knockdowns) on the fate of tryptophan or other medium components that potentially give rise to toxic byproducts. Future work should also investigate the impact of lowering the tryptophan concentrations in basal and/or feed media. A resultant improvement in cell growth would provide further supporting evidence that one or more products of tryptophan metabolism leads to growth inhibition. Recently, Mulukutla et al. reported that maintaining several amino acids at low concentrations throughout the culture duration improved both VCD and titer [8].

In conclusion, this study demonstrates the benefit of comparing multiple cell lines from different host lineages, as a metabolic indicator identified using this approach is more likely to reflect a general mechanism of growth inhibition. A similar approach could also be used to identify metabolic indicators of product quality, e.g., glycosylation. Prospectively, these cell line- and host-independent metabolic indicators could serve as biomarkers during clone selection and guide rational engineering of CHO cell hosts for improved growth and productivity.

## 4. Materials and Methods

### 4.1. Chemicals and Reagents

Unless otherwise noted, all chemicals and reagents, including LC–MS-grade water and other solvents, were purchased from Sigma Aldrich (St. Louis, MO, USA). Serum-free, chemically defined proprietary basal and feed media (described below) were prepared at Biogen.

### 4.2. Bioreactor Cell Culture

Six CHO cell lines, each producing different human monoclonal antibodies (IgG), were selected for metabolomics experiments. Three of the cell lines were derived from host 1 [28], a derivative of the K1 strain. This host was transfected with a single expression plasmid containing the heavy and light chain cassettes for each of the three antibodies of interest. Both heavy chain and light chain cDNAs were under the control of separate constitutive promoters. The GS gene (*GLUL*) was used as the selection marker. The *GLUL* selection marker was linked to the heavy chain cassette by an internal ribosome entry site element. The other three cell lines were derived from host 2 [29], a derivative of the DG44 strain. This host was independently transfected with an expression plasmid encoding mAb heavy chain and a second plasmid encoding light chains for each of the three antibodies of interest. The dihydrofolate reductase gene was used as the selection marker, and clones were selected with methotrexate-containing media using standard protocols. The hosts and cell lines were selected to investigate metabolite profiles of mAb-producing CHO cells with different growth characteristics. Combined with untargeted insertion of expression vectors, the above described differences between hosts have been shown to result in cell lines with significantly different genotypes and phenotypes [28].

All six clones were cultured in 5 L glass bioreactors (Applikon, Foster City, CA, USA) using TruBio DV controllers (Finesse Solutions, San Jose, CA, USA). Cryopreserved cells were thawed and scaled up in shake flasks (Corning, NY, USA) by passaging cultures every 3 to 4 days. Shake flasks were kept in a humidified incubator set at 36 °C and 5% CO_2_. Cells were counted using a viability analyzer (Vi-Cell, Beckman Coulter, Fullerton, CA, USA). Cells were inoculated in bioreactors at a seeding density of 1 × 10^6^ cells/mL for all cell lines except cell line 6 (derived from host 2). This cell line was seeded at a lower density (4 × 10^5^ cells/mL), because it did not achieve sufficiently high cell densities in seed flasks to match the inoculation density of the other cell lines. Serum-free, chemically defined proprietary basal and feed media and bioreactor operating conditions were used similar to what has been previously described [29]. The basal medium (CM3) was derived by supplementing a 1:1 mixture of Iscove’s Modified Dulbecco’s Medium (IMDM) and MCDB medium with amino acids, trace elements, and a non-ionic surfactant (Pluronic F68, ThermoFisher, Waltham, MA, USA). The feed medium (CF2b) was derived from a partial concentrate of CM3 by removing inorganic salts and additionally supplementing amino acids and growth factors. The bioreactors were fed every 24–48 h starting on day 3. The feeds were added based on integrated viable cell density (VCD), rather than a percentage of culture volume [3]. Supernatant samples were collected from the bioreactors during the exponential growth phase and the stationary phase and clarified by centrifugation followed by filtration, and then stored at −70 °C prior to metabolite extraction.

### 4.3. Sample Preparation

Previously frozen culture medium samples were thawed on ice and mixed with pure methanol at a 1:3 sample to methanol ratio (*v/v*). The mixture was vortexed for 15 sec and centrifuged at 15,000 × *g* for 15 min at 4 °C to pellet proteins. The supernatant was collected into a fresh sample tube and dried using a SpeedVac concentrator (Eppendorf Vacufuge 5301). The dried sample was reconstituted in one-half sample volume of methanol/water (1:1 *v/v*). The extraction process was repeated three times for each sample.

### 4.4. LC–MS Experiments and Feature Annotation

Cell culture supernatant samples were analyzed using information dependent acquisition (IDA) experiments on a time-of-flight (TOF) mass analyzer (AB SCIEX TripleTOF 5600+, Framingham, MA, USA) as previously described [18]. Details of the IDA experiments, including LC gradient methods (Tables S5 and S6), column specifications, and TOF instrument settings are provided in Supplementary Materials. Raw LC–MS data were preprocessed using XCMS [30], as previously described [18], to detect and align peaks. The peaks were analyzed using the CAMERA tool [31] to detect isotopes and adducts, and extract accurate masses. The resulting ion peaks were arranged into a feature table. Each peak in the table, or feature, is specified by a mass-to-charge (*m/z*) value and LC retention time (RT). Each feature is also associated with an MS/MS spectrum and an area under the curve (AUC) for the corresponding extracted ion chromatogram (XIC).

The features were annotated with five different tools: Metlin, HMDB, NIST MS Search 17, CFM-ID and MetFrag [32,33,34,35,36]. For many of the features, these tools returned different annotations. To determine the most likely identity for a feature in the context of CHO cell metabolism, we applied an automated annotation procedure that analyses the outputs of the aforementioned five tools in the context of a metabolic model for the biological system of interest [18]. A schematic (Figure S5) and description of the annotation procedure (‘BioCAn’) can be found in Supplementary Materials.

### 4.5. Supplementation Experiments in Shake Flask

To test whether excess tryptophan inhibits cell growth, we selected a cell line that achieved an intermediate peak VCD in the bioreactor study (cell line 4), and grew this cell line in shake flasks supplemented with different levels of tryptophan (5× and 10× basal level) in the basal medium. The cells were inoculated into shake flasks at a density of 2.5 × 10^5^ cells/mL and cultured in a humidified incubator set at 36 °C and 5% CO_2_. Each batch culture was run in duplicate. The cells were maintained in well-mixed suspension by placing the flasks on an orbital shaker set at 125 rotations per minute. Cells were counted manually by hemocytometer using trypan blue exclusion on days 0, 3, 6, and 9. Each manual count was repeated four times. Metabolites were extracted from culture medium samples and analyzed using LC–MS experiments, as described above.

### 4.6. Supplementation Experiments in Deep-Well Plate

To determine whether excess amino acids in the culture medium had a similar effect on multiple cell lines, all three cell lines from host 2 (cell lines 4, 5, and 6) and a new cell line from host 1 (cell line 7) were grown in culture media supplemented with one of 8 different amino acids (glycine, leucine, methionine, phenylalanine, serine, threonine, tryptophan, or tyrosine) at 10× their basal level in the basal medium. The cells were first expanded in shake flasks until the growth was exponential, and then transferred to a 24-deep well plate. Each well plate culture was seeded in duplicate at 2.5 × 10^5^ cells/mL. The plate was incubated with shaking in a humidified incubator set at 36 °C and 5% CO_2_. After 3 days, cells were counted on an automated cell viability analyzer. For each of the four cell lines, growth in basal medium was compared to growth in amino acid supplemented medium.

### 4.7. Statistics

All statistical calculations were performed in R (R Core Team, Vienna, Austria). Principal component analysis (PCA) was performed on mean-centered and autoscaled AUC data of features detected in samples collected from the bioreactors during the stationary phase [37]. Tests of significance on correlations or treatment effects are described in the relevant figure captions. The Benjamini–Hochberg (BH) procedure was applied to control the false discovery rate (FDR) for multiple comparisons. Tests were considered significant if the FDR-adjusted *p*-value was less than 0.05.

Pathway enrichment analysis was performed to identify pathways associated with metabolites that are significantly elevated in the slowest growing (Table S2) cell line (host 2, cell line 6) during the stationary phase relative to the other five cell lines (1–5). In addition to the slowest growth rate, cell line 6 also had the lowest VCD, which ensured that a metabolic byproduct measured at a higher concentration in the spent medium of cell line 6 is also elevated on a per cell basis. A one-sided t-test was performed to determine which feature responses (ion chromatogram AUC) were significantly elevated in samples for cell line 6 compared to the other cell lines. Features were considered significant if the FDR-adjusted *p*-values were less than 0.05. Each significant and annotated (putatively identified) feature was matched with associated KEGG pathways in the CHO model. The *p*-values for pathway enrichment analysis were determined by Fisher’s exact test using the R Stats Package function *fisher.test*. These *p*-values were then applied to contingency tables to determine whether a pathway was significantly enriched with metabolites elevated in cell line 6. The count of metabolites mapped to each pathway was reduced by one to provide a more conservative estimate of enrichment, similar to the EASE score [38] modification of Fisher’s exact test used in MetaboAnalystR [39]. A schematic of the contingency tables used for this test is shown in Supplementary Materials (Table S3).

Two-way analysis of covariance (ANCOVA) was applied to determine whether there were metabolites that accumulated in the bioreactors over time and correlated negatively with VCD. Growth stage was set as a categorical factor with two levels (exponential and stationary) and peak VCD was set as a continuous factor. One-way analysis of variance (ANOVA) was used to assess the effect of tryptophan on the day 3 growth rate in the supplementation experiments. The specific growth rate (µ) was calculated using the following equation:

(1) $$\mu_{day\;3}=\frac{\ln\left(VCD_{day\;3}-VCD_{day\;0}\right)}{3\;\mathrm{days}}.$$

Two-way ANOVA was used identify metabolites that accumulated in the shake flask cultures in response to increasing tryptophan concentration in the culture medium. The two factors were time in culture and level of tryptophan supplementation.

## Figures and Tables

**Table 1:** Pathway enrichment analysis.

Pathway	*p*-Value ^1^
Aminoacyl-tRNA biosynthesis	0.007
Tryptophan metabolism	0.030
Histidine metabolism	0.030

^1^ Controlled for false discovery rate (FDR) of multiple comparisons using the Benjamini–Hochberg procedure.

## Supplementary Materials

The following are available online at https://www.mdpi.com/2218-1989/10/5/199/s1, Figure S1: Heatmap of significant LC–MS features, Figure S2: Putatively identified histidine metabolite negatively correlating with cell growth, Figure S3: Tryptophan levels across cell lines, Figure S4: Effect of tryptophan supplementation on viable cell density and specific growth rate, Figure S5: Potential mechanism(s) of tryptophan metabolism-dependent growth inhibition in CHO cell culture, Figure S6: Effect of serotonin supplementation on day 3 viable cell density, Figure S7: Effect of indole 3-acetic acid on growth rate, Table S1: Putatively identified metabolites negatively correlated with cell growth, Table S2: Specific growth rate and average doubling time for cell lines in bioreactor culture, Table S3: Contingency table for pathway enrichment analysis, Table S4: Tryptophan metabolites elevated in the culture medium^1^ upon tryptophan supplementation, Table S5: Mobile-phase gradient for reverse-phase chromatography, and Table S6: Mobile-phase gradient for hydrophilic interaction chromatography.

## References

[1] Walsh G. (2014). Biopharmaceutical benchmarks 2014. Nat. Biotechnol..

[2] Wurm F.M. (2004). Production of recombinant protein therapeutics in cultivated mammalian cells. Nat. Biotechnol..

[3] Huang Y.-M., Hu W., Rustandi E., Chang K., Ryll T., Yusuf-Makagiansar H. (2010). Maximizing productivity of CHO cell-based fed-batch culture using chemically defined media conditions and typical manufacturing equipment. Biotechnol. Prog..

[4] Mulukutla B.C., Khan S., Lange A., Hu W.-S. (2010). Glucose metabolism in mammalian cell culture: New insights for tweaking vintage pathways. Trends Biotechnol..

[5] Gagnon M., Hiller G.W., Luan Y.-T., Kittredge A., DeFelice J., Drapeau D. (2011). High-End pH-controlled delivery of glucose effectively suppresses lactate accumulation in CHO Fed-batch cultures. Biotechnol. Bioeng..

[6] Pereira S., Kildegaard H.F., Andersen M.R. (2018). Impact of CHO Metabolism on Cell Growth and Protein Production: An Overview of Toxic and Inhibiting Metabolites and Nutrients. Biotechnol. J..

[7] Chong W.P., Yusufi F.N., Lee D.-Y., Reddy S.G., Wong N.S., Heng C.-K., Yap M.G., Ho Y.S. (2011). Metabolomics-based identification of apoptosis-inducing metabolites in recombinant fed-batch CHO culture media. J. Biotechnol..

[8] Mulukutla B.C., Kale J., Kalomeris T., Jacobs M., Hiller G.W. (2017). Identification and control of novel growth inhibitors in fed-batch cultures of Chinese hamster ovary cells. Biotechnol. Bioeng..

[9] Selvarasu S., Ho Y.S., Chong W.P.K., Wong N.S.C., Yusufi F.N.K., Lee Y.Y., Yap M.G.S., Lee D.-Y. (2012). Combined in silico modeling and metabolomics analysis to characterize fed-batch CHO cell culture. Biotechnol. Bioeng..

[10] Stolfa G., Smonskey M.T., Boniface R., Hachmann A.-B., Gulde P., Joshi A.D., Pierce A.P., Jacobia S.J., Campbell A. (2017). CHO-Omics Review: The Impact of Current and Emerging Technologies on Chinese Hamster Ovary Based Bioproduction. Biotechnol. J..

[11] Patti G.J., Yanes O., Siuzdak G. (2012). Innovation: Metabolomics: The apogee of the omics trilogy. Nat. Rev. Mol. Cell Biol..

[12] Dietmair S., Hodson M.P., Quek L.-E., Timmins N., Chrysanthopoulos P., Jacob S.S., Gray P., Nielsen L.K. (2012). Metabolite profiling of CHO cells with different growth characteristics. Biotechnol. Bioeng..

[13] Mohmad-Saberi S.E., Hashim Y.-Y., Mel M., Amid A., Ahmad-Raus R., Packeer-Mohamed V. (2012). Metabolomics profiling of extracellular metabolites in CHO-K1 cells cultured in different types of growth media. Cytotechnology.

[14] Chong W.P.K., Goh L.T., Reddy S.G., Yusufi F.N., Lee D.-Y., Wong N.S.C., Heng C.-K., Yap M.G.S., Ho Y.S. (2009). Metabolomics profiling of extracellular metabolites in recombinant Chinese Hamster Ovary fed-batch culture. Rapid Commun. Mass Spectrom..

[15] Sellick C.A., Croxford A.S., Maqsood A.R., Stephens G., Westerhoff H.V., Goodacre R., Dickson A.J. (2011). Metabolite profiling of recombinant CHO cells: Designing tailored feeding regimes that enhance recombinant antibody production. Biotechnol. Bioeng..

[16] Chong W.P.K., Thng S.H., Hiu A.P., Lee D.-Y., Chan E.C.Y., Ho Y.S. (2012). LC-MS-based metabolic characterization of high monoclonal antibody-producing Chinese hamster ovary cells. Biotechnol. Bioeng..

[17] Yeo H.C., Chen S., Ho Y.S., Lee D.-Y. (2018). An LC–MS-based lipidomics pre-processing framework underpins rapid hypothesis generation towards CHO systems biotechnology. Metabolomics.

[18] Alden N., Krishnan S., Porokhin V., Raju R., Mcelearney K., Gilbert A., Lee K. (2017). Biologically Consistent Annotation of Metabolomics Data. Anal. Chem..

[19] Hefzi H., Ang K.S., Hanscho M., Bordbar A., Ruckerbauer D., Lakshmanan M., Orellana C.A., Baycin-Hizal D., Huang Y., Ley D. (2016). A Consensus Genome-scale Reconstruction of Chinese Hamster Ovary Cell Metabolism. Cell Syst..

[20] King Z.A., Lu J., Dräger A., Miller P., Federowicz S., Lerman J., Ebrahim A., Palsson B.O., Lewis N.E. (2015). BiGG Models: A platform for integrating, standardizing and sharing genome-scale models. Nucleic Acids Res..

[21] Calmels C., McCann A., Malphettes L., Andersen M.R. (2019). Application of a curated genome-scale metabolic model of CHO DG44 to an industrial fed-batch process. Metab. Eng..

[22] A Rybaczyk L., Bashaw M.J., Pathak D.R., Huang K. (2008). An indicator of cancer: Downregulation of Monoamine Oxidase-A in multiple organs and species. BMC Genom..

[23] Soll C., Jang J.H., Riener M.-O., Moritz W., Wild P.J., Graf R., Clavien P.A. (2009). Serotonin promotes tumor growth in human hepatocellular cancer. Hepatology.

[24] Ke C., Hou Y., Zhang H., Fan L., Ge T., Guo B., Zhang F., Yang K., Wang J., Lou G. (2014). Large-scale profiling of metabolic dysregulation in ovarian cancer. Int. J. Cancer.

[25] Chiu J., Valente K.N., Levy N.E., Min L., Lenhoff A.M., Lee K.H. (2016). Knockout of a difficult-to-remove CHO host cell protein, lipoprotein lipase, for improved polysorbate stability in monoclonal antibody formulations. Biotechnol. Bioeng..

[26] Youdim M.B.H., Edmondson D., Tipton K.F. (2006). The therapeutic potential of monoamine oxidase inhibitors. Nat. Rev. Neurosci..

[27] Johnson C.H., Ivanisevic J., Siuzdak G. (2016). Metabolomics: Beyond biomarkers and towards mechanisms. Nat. Rev. Mol. Cell Boil..

[28] Wright C., Alves C., Kshirsagar R., Pieracci J., Estes S. (2017). Leveraging a CHO cell line toolkit to accelerate biotherapeutics into the clinic. Biotechnol. Prog..

[29] Gilbert A., Mcelearney K., Kshirsagar R., Sinacore M.S., Ryll T. (2013). Investigation of metabolic variability observed in extended fed batch cell culture. Biotechnol. Prog..

[30] Benton H.P., Want E.J., Ebbels T.M.D. (2010). Correction of mass calibration gaps in liquid chromatography–mass spectrometry metabolomics data. Bioinformatics.

[31] Kuhl C., Tautenhahn R., Böttcher C., Larson T.R., Neumann S. (2011). CAMERA: An Integrated Strategy for Compound Spectra Extraction and Annotation of Liquid Chromatography/Mass Spectrometry Data Sets. Anal. Chem..

[32] Allen F., Pon A., Wilson M., Greiner R., Wishart D.S. (2014). CFM-ID: A web server for annotation, spectrum prediction and metabolite identification from tandem mass spectra. Nucleic Acids Res..

[33] Ruttkies C., Schymanski E.L., Wolf S., Hollender J., Neumann S. (2016). MetFrag relaunched: Incorporating strategies beyond in silico fragmentation. J. Chemin..

[34] Wishart D.S., Jewison T., Guo A.C., Wilson M., Knox C., Liu Y., Djoumbou Y., Mandal R., Aziat F., Dong E. (2012). HMDB 3.0—The Human Metabolome Database in 2013. Nucleic Acids Res..

[35] Tautenhahn R., Cho K., Uritboonthai W., Zhu Z., Patti G.J., Siuzdak G. (2012). An accelerated workflow for untargeted metabolomics using the METLIN database. Nat. Biotechnol..

[36] Yang X., Neta P., Stein S. (2014). Quality Control for Building Libraries from Electrospray Ionization Tandem Mass Spectra. Anal. Chem..

[37] Berg R.V.D., Hoefsloot H., A Westerhuis J., Smilde A.K., Van Der Werf M.J. (2006). Centering, scaling, and transformations: Improving the biological information content of metabolomics data. BMC Genom..

[38] Hosack D.A., Dennis G., Sherman B.T., Lane H.C., Lempicki R.A. (2003). Identifying biological themes within lists of genes with EASE. Genome Biol..

[39] Chong J., Xia J. (2018). MetaboAnalystR: An R package for flexible and reproducible analysis of metabolomics data. Bioinformatics.

