# Supplementary material for: Using Metabolomics to Identify Cell Line-Independent Indicators of Growth Inhibition for Chinese Hamster Ovary Cell-Based Bioprocesses
Source: Metabolites. 2020 May 15;10(5):199. doi: 10.3390/metabo10050199 (PMC7281457; doi:10.3390/metabo10050199)
Supplement: Supplementary file 1 [file metabolites-10-00199-s001.pdf]

Using Metabolomics to Identify Cell Line-Independent Indicators of Growth Inhibition for Chinese Hamster Ovary Cell-based Bioprocesses

Nicholas Alden[1], Ravali Raju[2], Kyle McElearney[2], James Lambropoulos[2], Rashmi Kshirsagar[2], Alan Gilbert[2], Kyongbum Lee[1]*

[1] Tufts University, Department of Chemical and Biological Engineering, 4 Colby Street, Medford, MA, 02155, USA
[2] Biogen, 225 Binney St, Cambridge, MA, 02142, USA
* Correspondence: kyongbum.lee@tufts.edu

Untargeted LC-MS experiments

The information-dependent acquisition (IDA) experiments comprised a TOF MS (survey) scan and (triggered) high-resolution MS/MS (product ion) scans monitoring up to 4 candidate ions per cycle. The dependent scans were triggered whenever the survey scan detected a precursor ion with an exact mass in the range of m/z 40-1500. The mass tolerance was set to 50 mDa. The collision energy was set to 20 eV. Precursor ions having the same mass as a previously fragmented precursor ion were excluded from fragmentation for a 30 sec window to increase the probability of fragmenting different ions. Dynamic background subtraction was applied to select a precursor ion for fragmentation when its intensity is rising rapidly over several TOF scans. This improves detection of precursor ions, and results in MS/MS scans that are obtained at or near the corresponding precursor ions' maximal peaks in the chromatogram.

Chromatographic separation was performed using a binary pump HPLC system (1260 Infinity, Agilent). Each sample was run three times using different combinations of ionization modes and liquid chromatography methods. A reverse phase (RP) chromatography method was paired with negative mode electrospray ionization (ESI). The RP method used a C18 column (Synergi Hydro-RP 4μ 80Å 250 x 2.0 mm, Phenomenex, Torrance, CA). The column oven temperature was set to 15 °C. Solvent A was a 0.1% solution of formic acid in water (w/w), and solvent B was a 0.1% solution of formic acid in methanol (w/w). The mobile phase gradient is shown in Table S5. A hydrophilic interaction chromatography (HILIC) method was paired with both negative and positive mode ESI. The HILIC method used an aminopropyl column (Luna NH_2 5μ 100Å 250 x 2.0 mm, Phenomenex). The column oven temperature was set to 25°C. Solvent A was a 5% acetonitrile solution in water (v/v) with 20 mM ammonium acetate (pH 9.45). Solvent B was acetonitrile. The mobile phase gradient is shown in Table S6.

We used an automated workflow previously developed in our laboratory (reference 17 in the main text) to annotate the IDA data. Briefly, mass features detected by LC-MS experiments were mapped onto compounds in a metabolic model for CHO cells. The model was constructed based on KEGG Orthology identifiers (K numbers) and Enzyme Commission (EC) numbers associated with the Chinese hamster (*Cricetulus griseus*, organism code cge). These K and EC numbers were linked to KEGG reaction identifiers (R numbers). The reactions were then linked with their primary substrate-product pairs as defined by KEGG's RCLASS data.

The LC-MS features from the IDA experiments were mapped onto the model by matching their measured masses to the exact masses of compounds in the model. An Individual Score was computed for each mapped compound. This score sums the outputs (scaled from 0 to 1) from each

annotation tool (HMDB, NIST, CFM-ID and MetFrag). The mapped compounds were placed as nodes in a graph. An edge was drawn between two nodes if the corresponding compounds are a reactant-product pair in an enzymatic reaction per KEGG RCLASS data. Each compound node was assigned an Annotation Score that is the sum of Individual Scores of all other compounds within two reactions of the given node. The compound of a given mass with the highest Annotation Score was considered the most likely annotation for that mass.

Table S1. Putatively identified metabolites negatively correlated[1] with cell growth

KEGG ID[2]	Metabolite	Pathway
C00352	D-Glucosamine 6-phosphate	Alanine, aspartate and glutamate metabolism
C12270	N-Acetylaspartylglutamate	Alanine, aspartate and glutamate metabolism
C16591	3-Carbamoyl-2-phenylpropionic acid	Drug metabolism - cytochrome P450
C00439	N-Formimino-L-glutamate	Histidine Metabolism
C00450	(S)-2,3,4,5-Tetrahydropyridine-2-carboxylate	Lysine Degradation
C14805	1-Nitro-5-hydroxy-6-glutathionyl-5,6-dihydronaphthalene	Metabolism of xenobiotics by cytochrome P450
C01801	Deoxyribose	Pentose Phosphate Pathway
C00299	Uridine	Pyrimidine metabolism
C00637	Indole-3-acetaldehyde	Tryptophan Metabolism
C05634	5-Hydroxyindoleacetaldehyde	Tryptophan Metabolism
C00314	Pyridoxine	Vitamin B6 metabolism

[1] Metabolites in the table are increased at peak viable cell density (VCD) compared to the exponential growth phase for all six cell lines characterized in the bioreactor experiments. The metabolites are also elevated in the culture medium of cell line #6, which grew to the lowest peak VCD.
[2] KEGG ID refers to the LIGAND database's compound number.

Table S2. Specific growth rate[1] and average doubling time[2] for cell lines in bioreactor culture

Cell line (host)	Specific growth rate, day^{-1}	Average doubling time to peak VCD, days
**#1 (host 1)**	0.69	1.48
**#2 (host 1)**	0.72	1.22
**#3 (host 1)**	0.67	1.31
**#4 (host 2)**	0.54	1.85
**#5 (host 2)**	0.62	1.47
**#6 (host 2)**	0.51	3.02

[1] The specific growth rate for each cell line was calculated based on viable cell density (VCD) data for days 0 to 5 of bioreactor culture (6 time points). The VCD data for this period were log (base *e*) transformed and regressed with respect to time. The coefficient of determination and norm of residuals, respectively, ranged from 0.9984 and 0.0864 (cell line #4) to 0.9606 and 0.5797 (cell line #1).
[2] The average doubling time for each cell line was calculated by dividing the natural logarithm of 2 with the natural logarithm of peak to initial VCD ratio, and multiplying this result with the time it took for the cell line to reach its peak VCD.

Table S3. Contingency table[1] for pathway enrichment analysis

	**Higher at peak VCD[2] of low growth cell line**	**Not higher at peak VCD of low growth cell line**
**In pathway**	M	N
**Not in pathway**	O	P

[1]A contingency table was generated for every KEGG pathway associated with the Chinese hamster. Each cell holds the number of putatively identified (i.e., BioCAn annotated) metabolites belonging to one of four mutually exclusive categories. Cell M holds the number of metabolites that are significantly elevated in the culture medium of cell line #6 at peak VCD (i.e., stationary phase) compared to the other five cell lines and has membership in the pathway of interest. Cell N holds the number of metabolites that are not significantly elevated in the culture medium of cell line #6 and have membership in the pathway. Cell O holds the number of metabolites that are significantly elevated in the culture medium of cell line #6 and do not have membership in the pathway. Cell P holds the number of metabolites that are not significantly elevated in the culture medium of cell line #6 and do not have membership in the pathway. A modified Fisher's exact test using the EASE score was applied to the contingency table to determine if there is a significant association between the pathway and cell line #6, which had the lowest peak VCD of all cell lines characterized in the bioreactor experiments.
[2] VCD: viable cell density

Table S4. Tryptophan metabolites elevated in culture medium[1] upon tryptophan supplementation

**KEGG ID[2]**	**Metabolite**
C00637	Indole-3-acetaldehyde
C05653	Formylanthranilate
C05634	5-Hydroxyindoleacetaldehyde
C05635	5-Hydroxyindoleacetate
C00331	Indolepyruvate
C00078	L-Tryptophan
C00643	5-Hydroxy-L-tryptophan
C02700	L-Formylkynurenine

[1] Cell line # 4 from host 2 was cultured in shake flasks. The cells were grown in media containing different initial concentrations of tryptophan (1×, 5×, or 10× basal medium concentration). Metabolites accumulating in the culture medium with increasing tryptophan concentration were identified using two-way ANOVA. The two factors were initial tryptophan concentration and time in culture.
[2] KEGG ID refers to the LIGAND database's compound number.

Table S5. Mobile phase gradient[1] for reverse phase chromatography

Time (minutes)	Flow Rate (mL/min)	% Solvent B
0	0.2	3
8	0.2	3
38	0.2	95
45	0.2	95
47	0.2	3
55	0.2	3

[1] The solvent composition changed linearly over the indicated elution period.

Table S6. Mobile phase gradient[1] for hydrophilic interaction chromatography

Time (minutes)	Flow Rate (mL/min)	% Solvent B
0	0.3	85
15	0.3	0
28	0.3	0
30	0.3	85
60	0.3	85

[1] The solvent composition changed linearly over the indicated elution period.

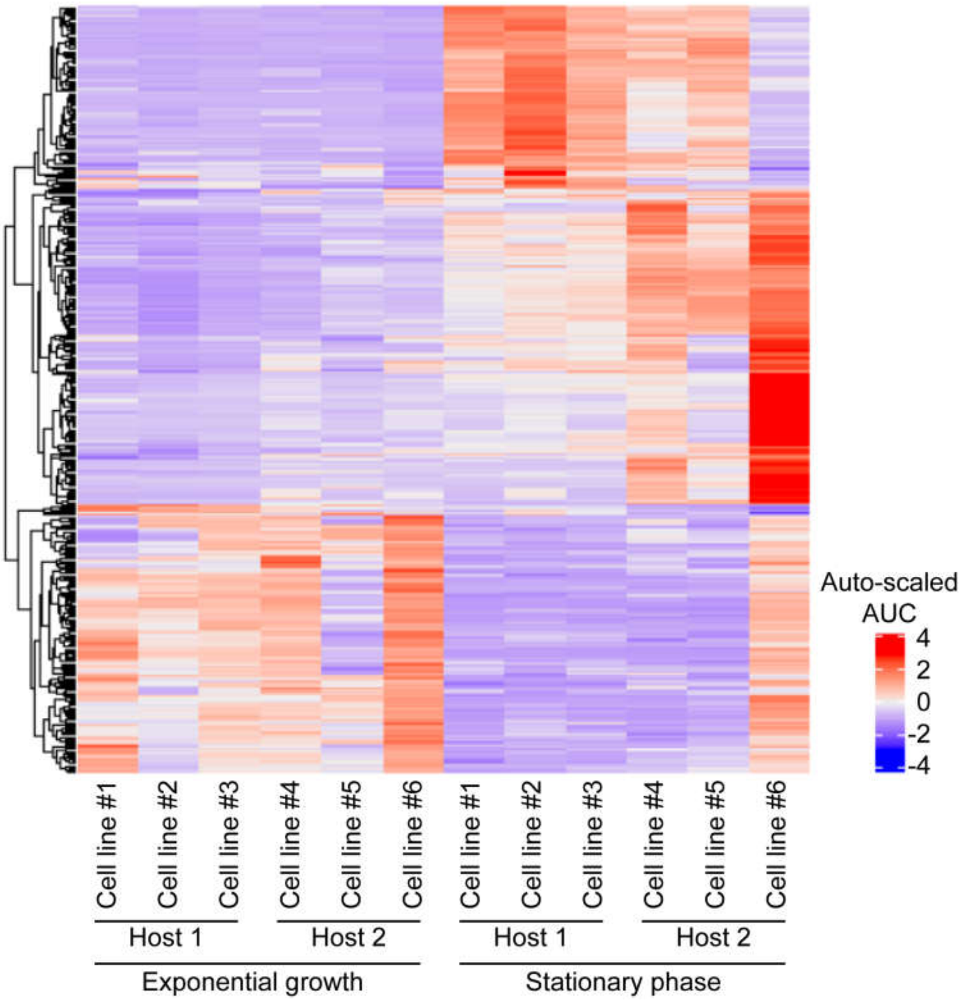

Figure S1. Heatmap of significant LC-MS features. Each row represents a unique feature detected in samples from all six cell lines of the bioreactor experiments. A total of 367 features increased in the culture medium at stationary phase compared to exponential growth phase across all six cell lines, while also correlating negatively with peak VCD. Data shown are averages of responses (areas-under-the-curve) from triplicate parallel sample extractions. Data were auto-scaled by row. The dendrogram shows results of k-means clustering.

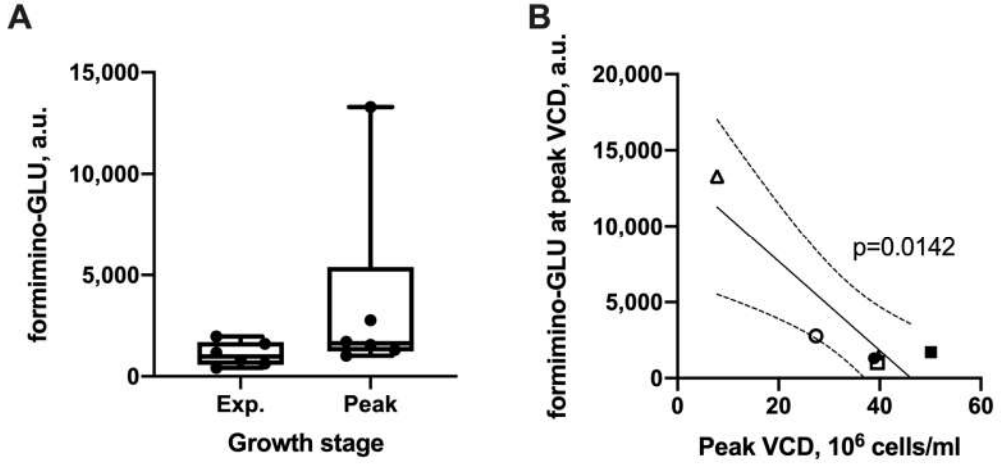

Figure S2. Putatively identified histidine metabolite negatively correlating with cell growth. (A) Medium concentrations of LC-MS feature putatively identified as N-formimino-L-glutamate (formimino-GLU) at exponential growth and stationary phase in the bioreactor cultures. Box plots show maximum, minimum, and median responses of the LC-MS feature (areas-under-the-curve in extracted ion chromatograms) across the six cell lines. (B) Scatter plots showing regression line and 95% confidence bands for formimino-GLU levels in the culture medium at peak VCDs of the six cell lines. Cell lines from host 1 (CHO K1 GS knockout) and host 2 (DG44) are indicated by closed and open symbols, respectively. A p-value < 0.05 indicates that the regression line slope is significantly different than zero by F-test.

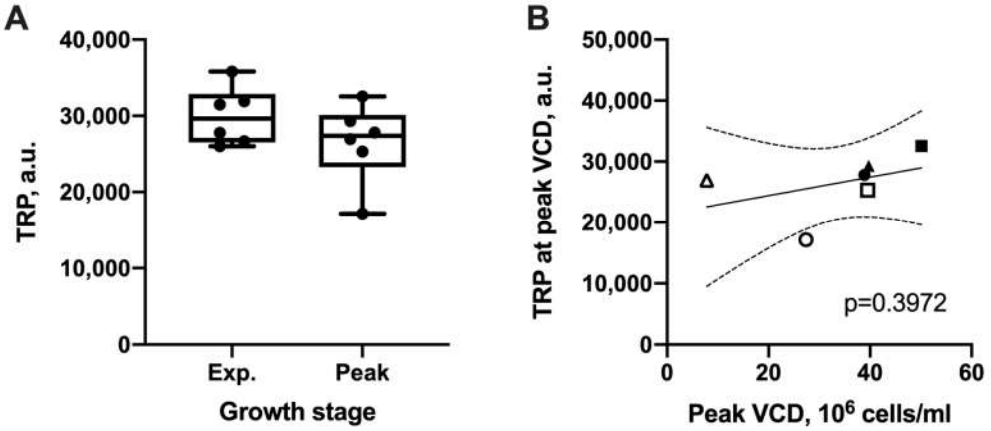

Figure S3. Tryptophan (TRP) levels across cell lines. (A) Medium concentrations of TRP at exponential growth and stationary phase in the bioreactor cultures. Box plots show maximum, minimum, and median responses of TRP across the six cell lines. Responses are integrated areas-under-the-curve in the extracted ion chromatograms. (B) Scatter plots showing regression line and 95% confidence bands for TRP levels in the culture medium at peak VCDs of the six cell lines. Cell lines from host 1 (CHO K1 GS knockout) and host 2 (DG44) are indicated by closed and open symbols, respectively. The regression line slope is not significantly different than zero by F-test.

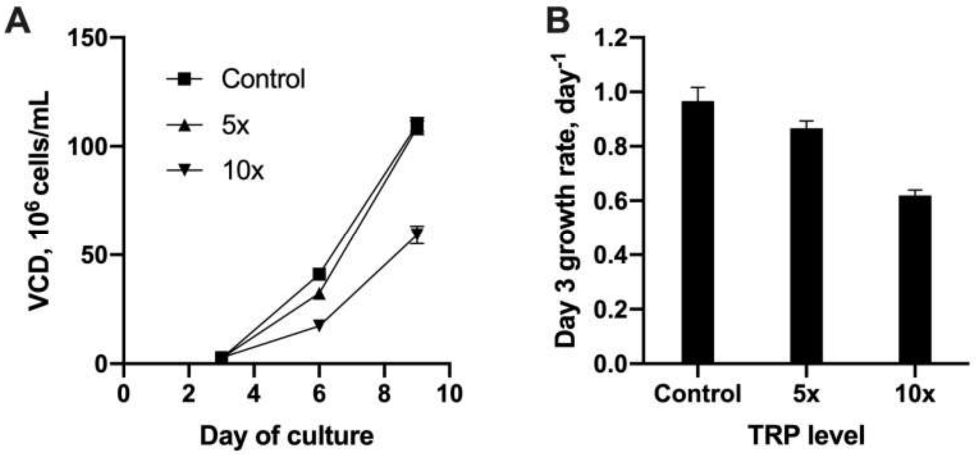

Figure S4. Effect of tryptophan (TRP) supplementation on (A) viable cell density (VCD) and (B) specific growth rate. Shake flasks were seeded with cell line #4 from host 2. The level of TRP supplementation above the basal medium concentration (Control) is indicated in the figure legend for panel (A) and x-axis labels for panel (B). Data shown are means ± SD of duplicate cultures. Some error bars are not visible, because they are smaller than plot symbols. The specific growth rate was calculated using equation (1) defined in the main text. The day 3 growth rate negatively correlates with the concentration of tryptophan in fresh culture medium (ANOVA p-value = 0.012).

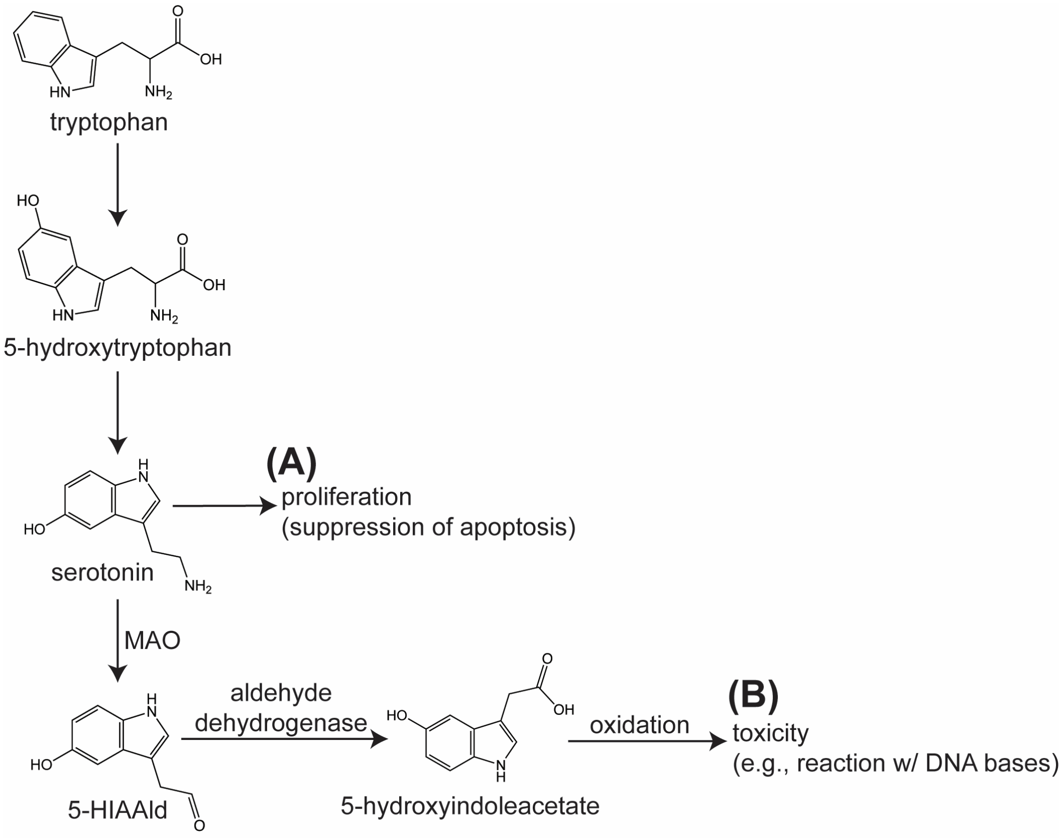

Figure S5. Potential mechanism(s) of tryptophan metabolism dependent growth inhibition in CHO cell culture. (A) Serotonin is a tryptophan metabolite that has been shown to increase proliferation of transformed cells by suppressing apoptosis (see main text for references). Monoamine oxidase (MAO) converts serotonin into 5-hydroxyindoleacetaldehyde (5-HIAAld). A high level of MAO activity could deplete serotonin, and reduce cell proliferation. (B) Alternatively, 5-HIAAld could directly inhibit growth, similar to other indole containing metabolites such as indole 3-acetate (IAA, Figure S8). The mechanism of IAA toxicity in CHO cells is unknown. In tumors, IAA toxicity is at least partially mediated by the formation of a reactive oxidation product, e.g., an oxindole [1].

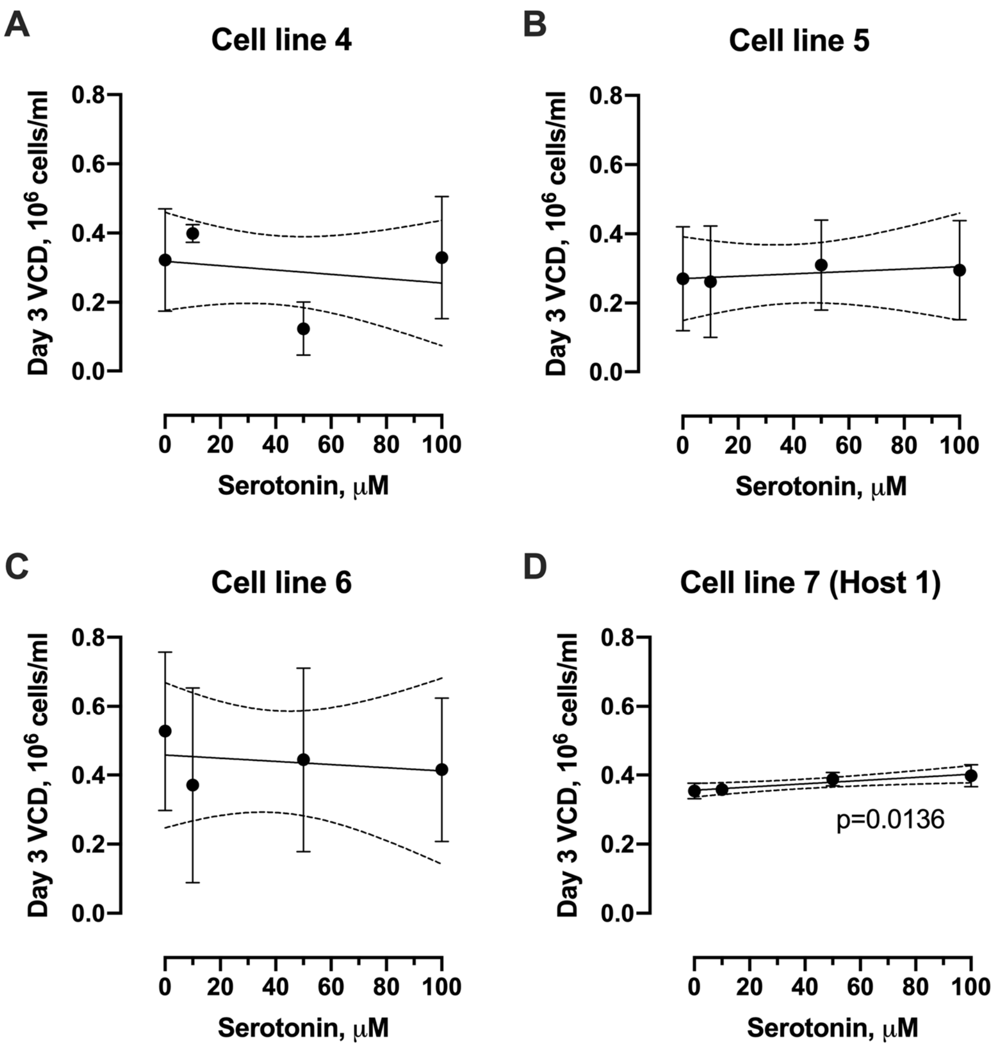

Figure S6. Effect of serotonin supplementation on day 3 viable cell density (VCD). Shake flasks were seeded with cell lines #4-6 (A-C) from host 2 and cell line #7 from host 1 (D). Data shown are means ± SD of triplicate cultures. Solid and dotted lines are regression line and 95% confidence bands, respectively. The regression line slope is not significantly different than zero by F-test for cell lines #4-6. A positive linear correlation between serotonin concentration and day 3 VCD was detected for cell line #7.

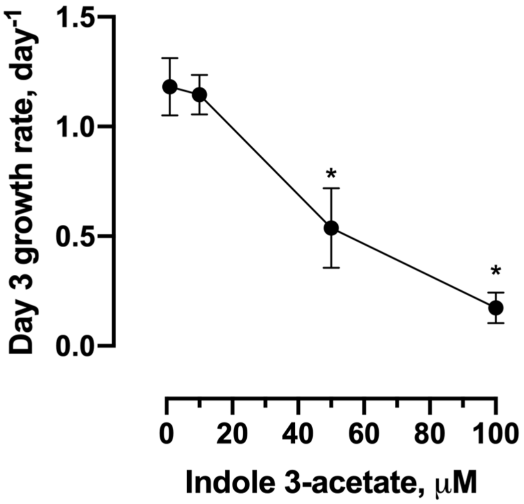

Figure S7. Effect of indole 3-acetic acid (IAA) on growth rate. Shake flasks were seeded with cell line #4 from host 2, which showed an intermediate growth rate in the bioreactor experiments. The specific growth rate was calculated using equation (1) defined in the main text. Asterisk (*) indicates a significantly lower specific growth rate compared to the control culture without any IAA supplementation (ANOVA $p < 0.05$).

References

1. Folkes, L.K.; Wardman, P. Oxidative activation of indole-3-acetic acids to cytotoxic species- a potential new role for plant auxins in cancer therapy. *Biochem Pharmacol* **2001**, *61*, 129-136, doi:10.1016/s0006-2952(00)00498-6.
